# Activated Lymphocytes and Increased Risk of Dermatologic Adverse Events during Sorafenib Therapy for Hepatocellular Carcinoma

**DOI:** 10.3390/cancers13030426

**Published:** 2021-01-23

**Authors:** Josep Corominas, Victor Sapena, Marco Sanduzzi-Zamparelli, Cristina Millán, Esther Samper, Neus Llarch, Gemma Iserte, Ferràn Torres, Leonardo G. Da Fonseca, Sergio Muñoz-Martínez, Alejandro Forner, Jordi Bruix, Loreto Boix, María Reig

**Affiliations:** 1 Barcelona Clinic Liver Cancer (BCLC) Group, Liver Unit, Hospital Clinic, IDIBAPS, University of Barcelona, 08036 Barcelona, Spain; jcorominas@clinic.cat (J.C.); vsapena@clinic.cat (V.S.); msanduzzi@clinic.cat (M.S.-Z.); cristinamdp.2020@gmail.com (C.M.); esamper@clinic.cat (E.S.); nllarch@clinic.cat (N.L.); giserte@clinic.cat (G.I.); l.fonseca@fm.usp.br (L.G.D.F.); smunozm@clinic.cat (S.M.-M.); aforner@clinic.cat (A.F.); jbruix@clinic.cat (J.B.); 2 Fundació Clínic Recerca Biomèdica, 08036 Barcelona, Spain; 3 Biostatistics Unit, Faculty of Medicine, Universitat Autònoma de Barcelona, 08193 Barcelona, Span; ferran.torres@uab.cat; 4 Medical Statistics Core Facility, Clinical Pharmacology Department, IDIBAPS-Hospital Clínic de Barcelona, 08036 Barcelona, Spain; 5 Centro de Investigación Biomédica en Red de Enfermedades Hepáticas y Digestivas (CIBERehd), 28028 Madrid, Spain

**Keywords:** hepatocellular carcinoma, sorafenib, early dermatologic adverse events, peripheral blood mononuclear cells, immune cell phenotyping, PD-1, DNAM-1, CD96

## Abstract

**Simple Summary:**

Hepatocellular carcinoma is the second cause of cancer-related death worldwide. Of those advanced-stage patients who are treated with sorafenib, those who develop early dermatologic adverse events have a better prognosis. These events are possibly immune-related. Therefore, we analyzed the phenotype of 52 sorafenib-treated patients’ circulating lymphocytes throughout treatment. We found that different co-stimulatory and immune exhaustion markers, such as Programmed cell death protein 1 (PD-1) and DNAX accessory molecule 1 (DNAM-1) amongst others, correlate with the probability of developing these adverse events, both before and during the treatment. We also compared the phenotype of those lymphocytes expressing DNAM-1 with those that do not, and while NK DNAM-1-expressing cells have a co-stimulatory phenotype, T DNAM-1-expressing cells are immune-suppressors. Overall, we set a rationale for the combination of sorafenib and immune-targeted therapies; and for the use of immune markers (such as DNAM-1) for patients’ prognosis evaluation.

**Abstract:**

Advanced hepatocellular carcinoma patients treated with sorafenib who develop early dermatologic adverse events (eDAEs) have a better prognosis. This may be linked to immune mechanisms, and thus, it is relevant to assess the association between peripheral immunity and the probability of developing eDAEs. Peripheral blood mononuclear cells of 52 HCC patients treated with sorafenib were analyzed at baseline and throughout the first eight weeks of therapy. T, B, Natural Killer cells, and their immune checkpoints expression data were characterized by flow cytometry. Cytokine release and immune-suppression assays were carried out ex vivo. Cox baseline and time-dependent regression models were applied to evaluate the probability of increased risk of eDAEs. DNAM-1, PD-1, CD69, and LAG-3 in T cells, plus CD16 and LAG-3 in NK cells, are significantly associated with the probability of developing eDAEs. While NK DNAM-1^+^ cells express activation markers, T DNAM-1^+^ cells induce immune suppression and show immune exhaustion. This is the first study to report an association between immune checkpoints expression in circulating immune cells and the increased incidence of eDAEs. Our results support the hypothesis for an off-target role of sorafenib in immune modulation. We also describe a novel association between DNAM-1 and immune exhaustion in T cells.

## 1. Introduction

Hepatocellular carcinoma (HCC) is the most common form of liver cancer (>80%) [1] and the second most common cause of cancer-related death worldwide [2]. Moreover, the overall burden of liver cancer is increasing over time. Up to 30–35% of the patients present advanced disease at diagnosis in countries without nationwide surveillance programs [3]. Currently, the combination of atezolizumab with bevacizumab has shown to be superior to sorafenib in first line [4], but not all HCC patients will be candidates for this option, and sorafenib and lenvatinib will remain the effective option for a relevant proportion of patients.

While having a main anti-angiogenic function, sorafenib has off-target activities on the immune system. It can enhance lymphocyte-mediated anti-tumor activity through an increase in lymphocyte infiltration [5,6], by targeting lymphocyte-specific protein tyrosine kinase (LCK) phosphorylation [7], and by inhibition of the major histocompatibility complex (MHC) shedding [8].

Early dermatologic adverse events (those arising in the first 60 days of treatment; eDAEs) predict a better outcome in HCC patients treated with sorafenib, with an overall survival of 18.2 months in patients with eDAEs vs. 10.1 months in patients without eDAEs [9]. The impact of eDAEs has been externally validated in sorafenib and regorafenib treated patients [10,11,12]. Skin adverse events have also been associated with better outcomes in other tumor indications treated with immune checkpoint inhibitors (ICIs) such as atezolizumab [13,14,15].

The underlying molecular mechanisms of eDAEs development, how they are triggered, and how these mechanisms contribute to a better response to sorafenib, are questions that have yet to be elucidated.

The advent of immune checkpoint inhibition therapies has revolutionized the landscape of cancer treatments over the last decade. These therapies are based on the use of monoclonal antibodies directed against inhibitory checkpoints, expressed by immune cells; or against their ligands, expressed by tumor cells. The disruption of these receptor/ligand interactions may revert the functional inhibition of these cells and restore an effective anti-tumor activity [16]. Since the discovery of Cytotoxic T-Lymphocyte Antigen 4 (CTLA-4) and Programmed Cell Death Protein 1 (PD-1) as therapeutic targets, the list of checkpoints has been increasing, and numerous efforts are focused on the discovery of new molecules that regulate the dynamics of the immune system [17]. Three PD-1 inhibitors (nivolumab, pembrolizumab, and cemiplimab) and three PD-1-Ligand inhibitors (atezolizumab, avelumab, and durvalumab) are in the current list of agents approved by the FDA for the treatment of a wide range of malignancies [18]. In the case of HCC, nivolumab and pembrolizumab are approved for second-line treatment after sorafenib.

Lymphocyte Activation Gene-3 (LAG-3) is another checkpoint marker associated with the inhibition of effector T cells and the promotion of regulatory T cells (Treg) through crosslinking with CD3, which inhibits T cell proliferation, cytokine production, and calcium flux [19].

Although initially described for lymphocyte T cells, further research has shown that other immune populations like Natural Killer (NK) cells also have their own set of checkpoint molecules. Some of these checkpoints prevent the activation of NK cells against healthy tissues, whilst others work to overcome the inhibition and elicit cytotoxic responses. The Natural Killer Group 2 member NKG2A recognizes the Human Leukocyte Antigen (HLA) and inhibits the NK cell response, whilst another member, NKG2D, recognizes induced self-proteins commonly expressed on stressed or malignant cells activating the NK cell response [20].

Low-Affinity IgG Fc Receptor Region Receptor III (CD16) is the most potent activating receptor expressed by NK cells [21]. Upon IgG-induced crosslinking of CD16 NK cells engage on antibody-dependent cell-mediated cytotoxicity, an adaptive immunity-like mechanism.

Tactile (CD96), the T Cell Immunoreceptor with Ig and ITIM Domains (TIGIT) and DNAX Accessory Molecule 1 (DNAM-1) share the common ligands Poliovirus receptor (CD155) and Nectin-2 (CD112) and are part of the group of Immunoglobulin-superfamily members [22]. While DNAM-1 has been shown to boost NK cytotoxic responses, TIGIT and CD96 are thought to act as a counter-balance to DNAM-1 and inhibit the cytotoxic response by competitive binding against their common ligands [23].

Metabolic pathways can also contribute to immune cell regulation in cancer. During inflammation, extracellular ATP undergoes phosphohydrolysis by ectonucleotidases (most prominently NTPDase 1, also known as CD39), culminating in the formation of high levels of adenosine within the tumor microenvironment (TME). Adenosine is an immunosuppressive metabolite that regulates tumor immunity, and targeting its pathway may provide therapeutic benefit [24].

Immune checkpoints are not restricted to one specific cell type, but rather can be expressed in more than one cell population depending on the physiologic conditions or the anatomic compartment [25,26]. This is the case for PD-1 and DNAM-1, which are also expressed in NK and T cell populations, respectively [27,28]. However, despite its co-stimulatory function in NK cells and cytotoxic T CD8^+^ cells, the role of DNAM-1 in other immune cells is not yet clear.

Amongst the transcription factors that dictate the functional programs of immune cells, Eomesodermin (Eomes) and T-box Protein 21 (T-bet) have emerged as crucial for the development and maturation of lymphocytes, where they tip the balance between effector/memory/tolerant functions [29,30,31,32].

In this study, we have investigated the distribution and functional status of peripheral blood immune B, T, and NK lymphocyte populations of patients developing eDAEs under sorafenib treatment. Our results show that the expression of DNAM-1 and PD-1 on T cells, and in less measure, the expression of CD16 and LAG-3, correlate with the probability of eDAEs appearance. Further characterization of PBMCs DNAM-1^+^ reveals its association with CD96, TIGIT, and T-bet. Overall, the results confirm that the immune system plays a role in sorafenib-associated eDAEs and provide a new rationale for sorafenib plus ICI therapy combination.

## 2. Results

### 2.1. Patients

A total of 52 patients were recruited for the study (Table 1). A median of 4 samples were extracted from every patient. Forty-four patients were men (84.62%) and 8 (15.38%) women, with a median age of 64.34 years. Twenty-two patients were staged as BCLC B (42.31%) and 30 (57.69%) as BCLC C, while 42 (80.77%) were Child-Pugh A and 7 (13.46%) were Child-Pugh B. Performance Status was 0 for 50 patients (96.15%) and 1 for 2 patients (3.85%). Twenty patients (38.46%) had vascular invasion and 22 (42.31%) had extrahepatic spread.

Median sorafenib treatment duration was 5.1 months [2.5–9.6], the median follow-up was 9.6 months [3.9–19.2] and patients’ median overall survival was 26.4 months (95% CI: 10.7–42.2).

Sixteen patients (30.8%) developed early dermatologic adverse events requiring dose modification, with a median overall survival of 26.4 months (95% CI: 17.4–35.5). Non-eDAEs patients had a median overall survival of 16.4 months (95% CI: 6.6-NE).

### 2.2. The Increase of B Cell Population during Sorafenib Treatment Increases the Probability of Developing eDAEs

No lymphocyte population at baseline was associated with eDAEs development. To assess whether changes throughout the treatment, rather than the baseline values, are responsible for the eDAEs, we used Cox regression models generated with time-dependent data. These models consider the evolutionary values of the selected variables at each recorded time during the first 8 weeks of treatment, allowing us to take into account the changes of specific variables over time and accurately assess their influence on the outcome. These models show that an increase in B cells correlates with higher probability of developing eDAEs (Table 2). The correlation, although not statistically significant, between an increase in NK cells (Table 2) and a lower probability of developing eDAEs is also found. No association was revealed between T cells or any of its subpopulations and the probability of developing eDAEs in neither of these models.

### 2.3. Lymphocytes Expressing DNAM-1 and PD-1 Are Significantly Associated with the Probability of Developing eDAEs, Both in Baseline and Time-Dependent Models

We next considered whether the lymphocyte’s immune checkpoint expression could lead to the development of eDAEs. With this purpose in mind, we determined the number of cells positive for the immune checkpoints PD-1 and LAG-3, which act as immune suppressors; DNAM-1 and NKG2D, activators of the NK cell cytotoxic response; and CD16, which triggers the NK antibody-dependent cell-mediated cytotoxicity. We also assessed the expression of CD39, the rate-limiting enzyme in the conversion of ATP to immunomodulatory adenosine; of CD69, a membrane-bound, type II C-lectin receptor that marks early activation of different immune subsets [33]; and of the IL-7 receptor subunit-α (CD127), associated with chronic inflammation and poorer outcome in cancer patients [34].

**Figure 1:**
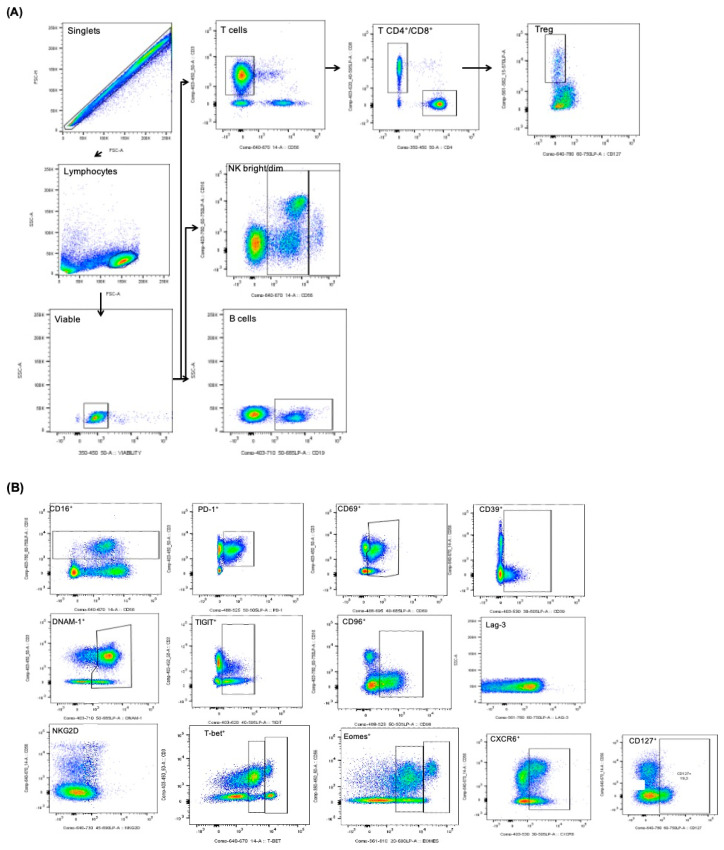
Lymphocytes gating strategy. (**A**) Flow chart depicting the order for subsetting the different lymphocyte populations. Events were initially gated on single events, then on lymphocytes by size and scatter. Living cells were gated next. From there, the different populations were gated based on their markers. (**B**) Gating strategy for the different lymphocyte immune checkpoints. Representative examples for the gatings of the different checkpoints used in the study. LAG-3 and NKG2D could not be gated and were analyzed by MFI quantification instead. T CD4^+^: T helper cells, T CD8^+^: cytotoxic T cells, NK: Natural Killer cells, Treg: regulatory T cells.

Figure 1 shows representative FACS dot plots to illustrate the gating strategy for all cell populations and cell markers analyzed in our study.

Cox regression models using baseline information identified that T lymphocytes, either CD4^+^ or CD8^+^, expressing DNAM-1 (DNAM-1^+^) correlated with a lower probability of developing eDAEs (Table 3). The same association was demonstrated for those NK cells with highest CD56 intensity (CD56^+bright^) expressing PD-1 (PD-1^+^) (Table 3). In contrast, there was a significant relationship between T CD8^+^ cells expressing CD69 (CD69^+^) and a higher probability of developing eDAEs (Table 3).

Time-dependent models also confirmed that the increase of T cells, either CD4^+^ or CD8^+^, expressing DNAM-1 concurred with lower eDAEs probability (Table 3). The same applies for the increase of T CD4^+^ cells expressing PD-1 (Table 3). This model also revealed that the increase of NK CD56^+bright^ cells expressing DNAM-1 or PD-1 was associated with a lower probability of developing eDAEs (Table 3).

### 2.4. CD16, PD-1, and LAG-3 Are Co-Expressed in NK-Like CD3^+^ Cells and Correlate with eDAEs Development

Further analysis of the time-dependent Cox regression models showed that the increase over time of NK-like CD3^+^ cells expressing CD16 (CD16^+^) correlated with a higher probability of developing eDAEs (Table 3). The same effect was found for NK-like CD3^+^ cells with higher quantification of PD-1 and LAG-3 measured by Mean Fluorescence Intensity (MFI). Since in NK and NK-like CD3^+^ cells LAG-3 expression could not be qualitatively distinguished due to its low levels, MFI quantification was undertaken instead.

As stated above, the increase of T cells expressing PD-1 correlated with a lower probability of developing eDAEs, while the increase of NK-like CD3^+^ cells expressing PD-1 correlated with a higher probability. Since the latter association is also found in NK-like CD3^+^ cells expressing CD16 or LAG-3, we evaluated whether the co-expression of PD-1 with CD16 and LAG-3 could explain the opposite results between NK-like CD3^+^ PD-1^+^ and T PD-1^+^ cells effects on eDAEs probability.

**Figure 2:**
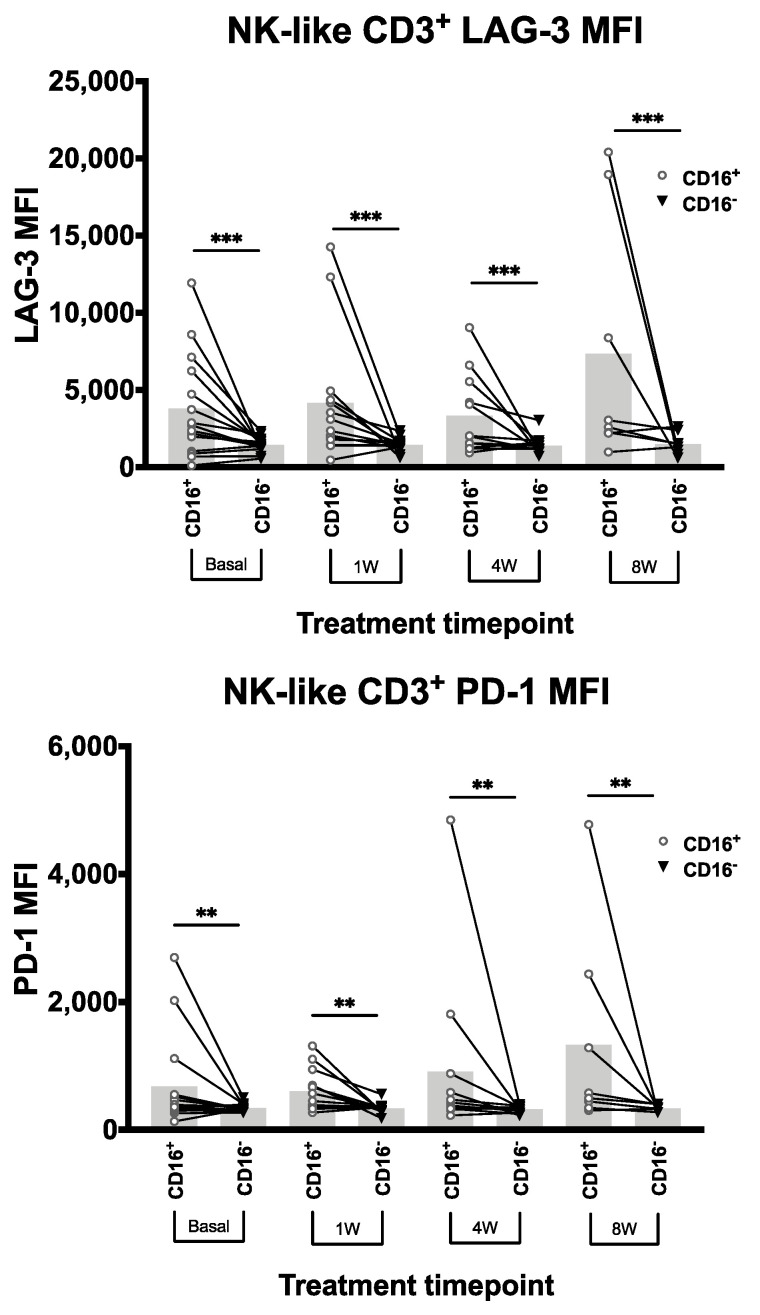
PD-1 and LAG-3 cluster with CD16 in NK-like CD3^+^ cells of patients developing eDAEs. LAG-3 (**top**) and PD-1 (**bottom**) Mean Fluorescence Intensity (MFI) quantification in NK-like CD3^+^ cells with and without CD16 expression (CD16^+^ and CD16^−^ respectively) at baseline and 1, 4, and 8 weeks (*n* = 15). Bar height represents the mean of each group. MFI: Mean Fluorescence Intensity. **: *p* < 0.01, ***: *p* < 0.005.

In the first subset of patients (*n* = 15), we found that NK-like CD3^+^ cells expressing CD16 had higher PD-1 and LAG-3 expression measured by MFI at all timepoints of the first 8 weeks (Figure 2), suggesting that they are co-expressed (834.15 mean PD-1 MFI and 4110.64 mean LAG-3 MFI in CD16^+^ cells, versus 335.35 mean PD-1 MFI and 1424.17 mean LAG-3 MFI in CD16^−^ cells).

To assess how this co-expression changes over time, we used a correlation analysis including the whole set of patients and found a positive association for CD16, LAG-3, and PD-1 in those patients who develop eDAEs, while a negative relationship was found for those with no eDAEs (Table 4).

### 2.5. T DNAM-1^+^ Cells Have an Exhausted Phenotype Compared to DNAM-1^−^ Counterparts; While NK DNAM-1^+^ Cells Have an Immune Activated Phenotype

**Figure 3:**
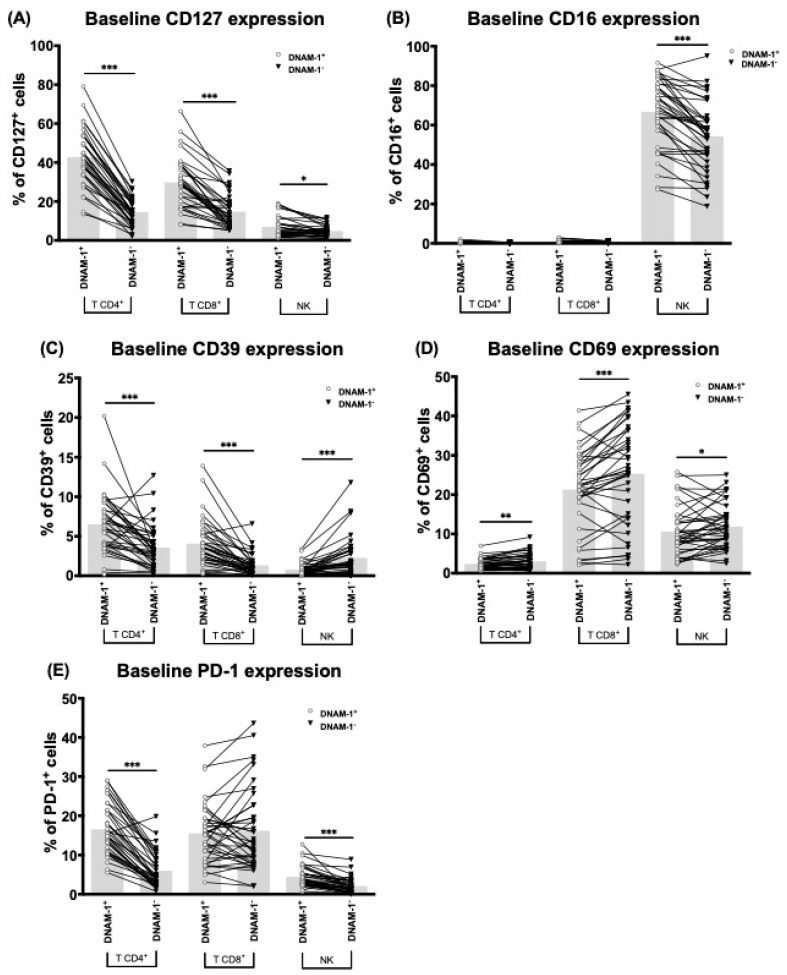
DNAM-1^+^ cells characterization. Comparison between CD127 (**A**), CD16 (**B**), CD39 (**C**), CD69 (**D**), and PD-1 (**E**) expressing cells in DNAM-1^+^ and DNAM-1^−^ subgroups of T CD4^+^, T CD8^+^, and NK cells of sorafenib treated patients at baseline (*n* = 52). Bar height represents the mean of each group. *: *p* < 0.05, **: *p* < 0.01, ***: *p* < 0.005.

After determining the immune checkpoints associated with the development of eDAEs, we assessed the role of DNAM-1 in T cells and in NK cells. To this end, we compared the phenotype of DNAM-1-expressing cells (DNAM-1^+^) against cells without DNAM-1 expression (DNAM-1^−^) in each patient using only the baseline samples. We compared the number of cells expressing PD-1 (PD-1^+^), CD127 (CD127^+^), CD39 (CD39^+^), CD69 (CD69^+^), and CD16 (CD16^+^) in each group and quantified the MFI of all these markers plus LAG-3 and NKG2D, whose expression could not be qualitatively distinguished. As previously described, PD-1^+^, CD127^+^, and CD39^+^ cells were more prevalent in T cells compared to NK cells, while CD16^+^ cells were only found in NK cells. CD69^+^ cells were found in both T CD8^+^ and NK cells (Figure 3).

T DNAM-1^+^ cells contained a higher number of CD127^+^, PD-1^+^ and CD39^+^ cells and lower numbers of CD69^+^ cells than T DNAM-1^−^ cells (Figure 3A–E). No difference was seen in NK cells for CD127, PD-1, and CD69 expression (Figure 3A,D,E). However, unlike T cells, there were fewer CD39^+^ cells in the DNAM-1^+^ group than in the DNAM-1^−^ group (Figure 3C). In the case of CD16^+^ cells, these were increased in the DNAM-1^+^ group compared to the DNAM-1^−^ group (Figure 3B).

**Figure 4:**
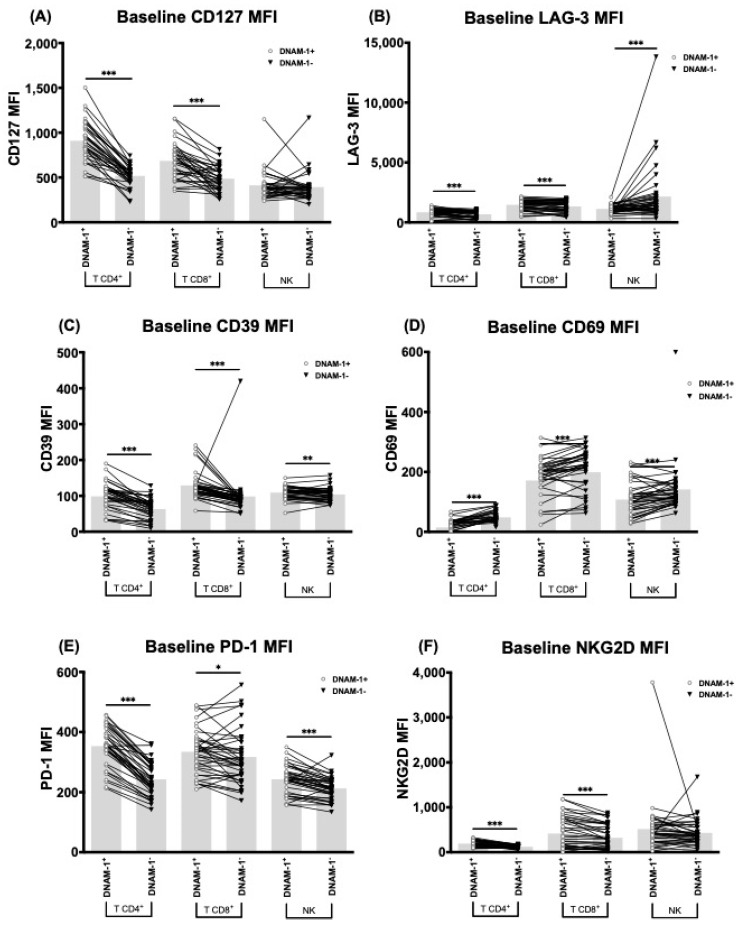
DNAM-1^+^ cells characterization. Comparison between CD127 (**A**), LAG-3 (**B**), CD39 (**C**), CD69 (**D**), PD-1 (**E**), and NKG2D (**F**) MFI quantification in DNAM-1^+^ and DNAM-1^−^ subgroups of T CD4^+^, T CD8^+^, and NK cells of sorafenib treated patients at baseline (*n* = 52). Bar height represents the mean of each group. MFI: Mean Fluorescence Intensity. *: *p* < 0.05, **: *p* < 0.01, ***: *p* < 0.005.

CD127, LAG-3, CD39, CD69, PD-1 and NKG2D MFI quantification allowed us to confirm the results and define additional differences for LAG-3 and NKG2D expression (Figure 4). For T cells, both LAG-3 and NKG2D had higher MFI in the DNAM-1^+^ group than in the DNAM-1^−^ group (Figure 4B,F). In NK cells, LAG-3 showed the opposite trend, with lower expression in DNAM-1^+^ cells than in the DNAM-1^−^; and no differences were found for NKG2D (Figure 4B,F).

### 2.6. CD96, an NK Inhibitory Checkpoint, Is Highly Expressed in T and NK-Like CD3^+^ Cells, But Not in Conventional NK Cells

DNAM-1 is part of the Ig-like receptor family together with CD96 and TIGIT, which share the common ligands Nectin-2 (CD112) and PVR (CD155). Eomes and T-bet are key transcription factors that regulate the process of lymphocytes maturation. To further characterize the phenotype of DNAM-1-expressing lymphocytes, we analyzed the distribution of CD96^+^, TIGIT^+^, Eomes^+^ and T-bet^+^ cells in the DNAM-1^+^ and DNAM-1^−^ groups using a subset (*n* = 8) of our patients’ baseline samples.

**Figure 5:**
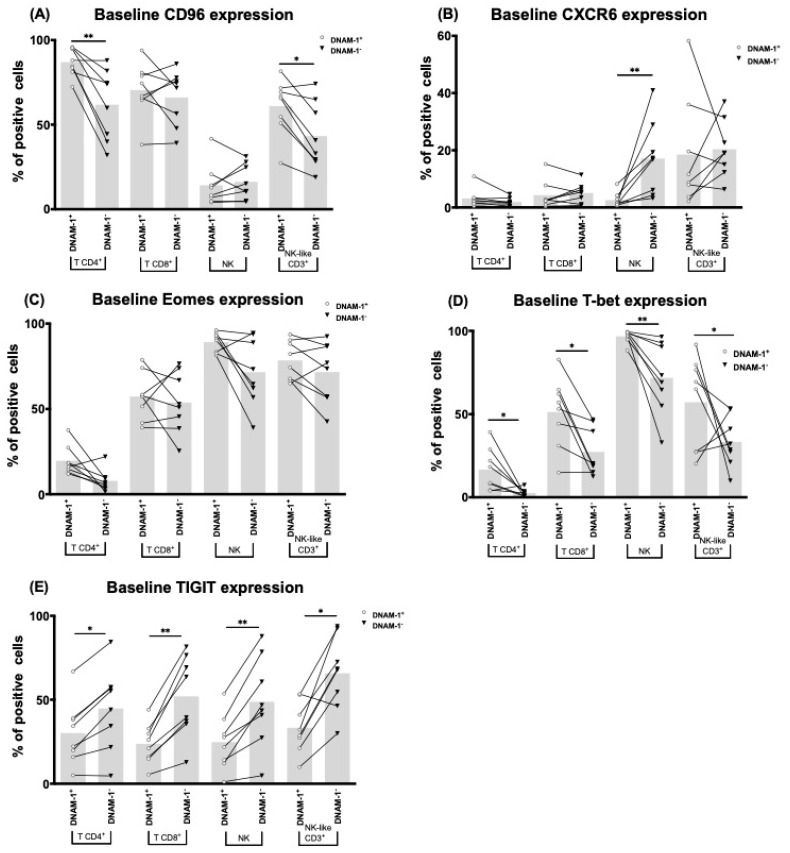
DNAM-1^+^ cells characterization. CD96 (**A**), CXCR6 (**B**), Eomes (**C**), T-bet (**D**), and T Cell Immunoreceptor with Ig and ITIM Domains (TIGIT) (**E**) positive cells across the DNAM-1^+^ and DNAM-1^−^ subgroups of T and NK cells of sorafenib treated patients at baseline (*n* = 8). Bar height represents the mean of each group. *: *p* < 0.05, **: *p* < 0.01.

Although being widely described as an NK inhibitor, our results show that CD96^+^ cells are more prevalent in T and NK-like CD3^+^ cells compared to NK cells (Figure 5A). When comparing DNAM-1^+^ and DNAM-1^−^ cells, CD96^+^ cells were more abundant in the T DNAM-1^+^ and NK-like CD3^+^ DNAM-1^+^ cells (Figure 5A), but no difference was found in NK cells (Figure 5A).

TIGIT^+^, Eomes^+^, and T-bet^+^ cells were found across all lymphocyte subtypes, and like CD96 they showed a different distribution amongst DNAM-1^+^ and DNAM-1^−^ groups. For TIGIT and T-bet, more T-bet^+^ cells and fewer TIGIT^+^ cells were found in the DNAM-1^+^ group, regardless of the lymphocyte subtype (Figure 5D,E).

On the other hand, different levels of Eomes^+^ cells were found in T CD4^+^ and CD8^+^ DNAM-1^+^/DNAM-1^−^ cells. T CD4^+^ DNAM-1^+^ cells contained more Eomes^+^ cells, while T CD8^+^ DNAM-1^+^ cells contained fewer Eomes^+^ cells (Figure 5C).

Finally, CXCR6, an NK marker associated with liver residency and considered residual in circulating lymphocytes, was found significantly expressed in NK-like CD3^+^ cells and in NK DNAM-1^−^ cells (Figure 5B).

### 2.7. T CD4^+^ DNAM-1^+^ Cells Present Higher Immune-Suppressive Ability Ex Vivo

**Figure 6:**
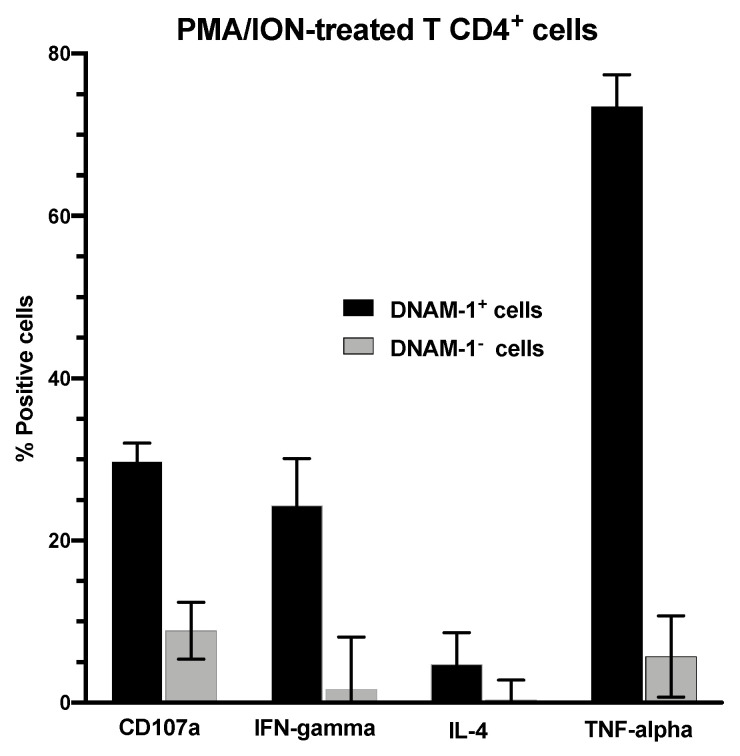

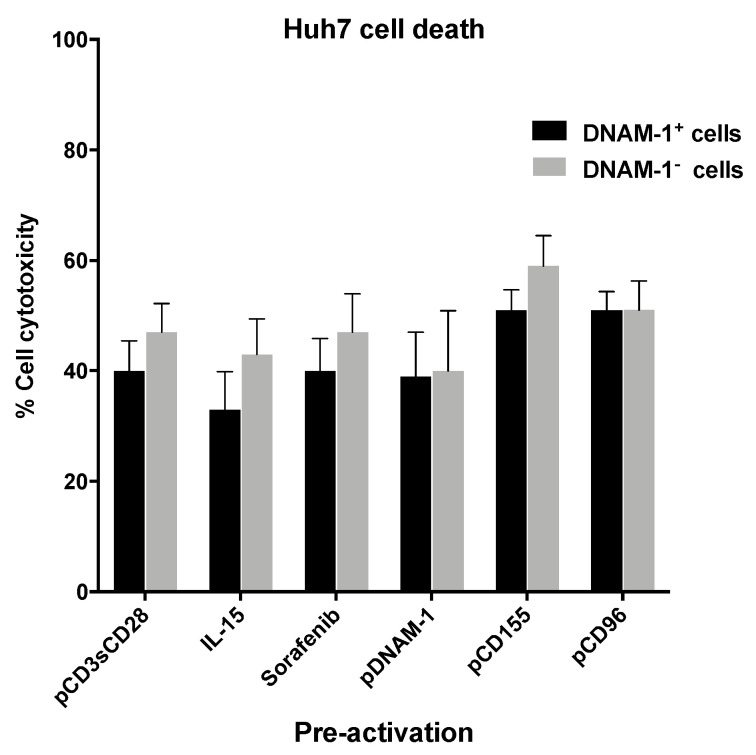
Effects of activated T CD4^+^ DNAM-1^+^ and DNAM-1^−^ cells on cytotoxic response. **Top**: Human T CD4^+^ cells were isolated from buffy coat and stimulated with PMA (50 ng/mL) and ionomycin (1 µg/mL) for 6 h in presence of Brefeldin-A (500 ng/mL) and anti-CD107a antibody. At the end of stimulation cells were fixed, permeabilized, and stained for quantification of CD107a, IFN-γ (gamma), IL-4 and TNF-α (alpha). **Bottom**: Cytotoxic capacity of effector CD4^−^ lymphocytes co-cultured with activated T CD4^+^ DNAM-1^+^ and DNAM-1^−^ cells. T CD4^+^ cells were pre-activated for 72 h before being moved into culture with autologous lymphocytes and Huh7 cells. After 24 h, cytotoxicity was assessed using MTS viability assay. PMA: phorbol myristate acetate, ION: Ionomycin, pCD3: plate-bound anti-CD3, sCD28: soluble anti-CD28, Sora: Sorafenib, pDNAM-1: plate-bound anti-DNAM-1, pCD155: plate-bound anti-CD155, pCD96: plate-bound anti-CD96.

To confirm the functional differences between T CD4^+^ DNAM-1^+^ and DNAM-1^−^ cells, we isolated both populations from blood of healthy individuals and stimulated them with PMA/ION to assess their cytokine release pattern. DNAM-1^+^ cells had higher expression of CD107a, IFN-γ, IL-4, and TNF-α (Figure 6), with TNF- α presenting the highest change of the four. No difference was found in IL-10, Grnz-B, and CD69 expression (Figure S1). Stimulation with other different factors did not render any differences in the cytokine expression (Figure S2).

Since PMA/ION stimulation is highly unspecific, we next evaluated how both populations specifically regulate the anti-tumor response. Pre-stimulated T CD4^+^ DNAM-1^+^ and DNAM-1^−^ cells were co-cultured with autologous CD4^−^ effector lymphocytes and HuH7 hepatocarcinoma cells. After 24 h of co-incubation, we found that those effector lymphocytes co-cultured with T CD4^+^ DNAM-1^+^ cells had less cytotoxic capacity compared to those co-cultured with DNAM-1^−^ cells (Figure 6). This effect was only seen at a T CD4^+^ cell 2:1 ratio, compared to 1:1, 1:2, and 1:10 ratio (Figure S3). Of note, sorafenib and plate-bound CD96 induced higher cytotoxicity than the other factors.

## 3. Discussion

This is the first study assessing the relevance of circulating immune cells in the development of early dermatological adverse events under sorafenib treatment for HCC. Our group has previously reported that eDAEs are tightly associated with a better patient outcome [10,11], and our data strongly reinforces the immune basis for such an association. Thus, the survival benefit of tyrosine kinase inhibitors (in this case, sorafenib) is in part mediated by the modulation of the immune system through off-target activity.

We demonstrated a significant association between the increase in B and NK cells during treatment (but not at baseline) and the probability of developing eDAEs. Specifically, we observed that an increase of B cells is associated with higher eDAEs probability while an increase of NK cells correlates with lower probability. These results highlight that a treatment-related change in lymphocyte populations, rather than their baseline levels, could be the responsible for eDAEs development.

To further characterize the changes in the immune populations of sorafenib-treated patients, we evaluated whether the lymphocytes phenotype changed in the course of therapy. We analyzed a panel of immune checkpoints, both inhibitory and co-stimulatory, across the different lymphocyte subpopulations. Interestingly, we found a significant association between the expression of DNAM-1, PD-1, LAG-3, CD69, and CD16 and the development of eDAEs.

DNAM-1 (or CD226) was first described as an immune checkpoint expressed in NK cells as part of the CD96/TIGIT/DNAM-1 Ig-like family [22,23,35,36], which share an ITIM domain that is recognized by the common ligand PVR (CD155). DNAM-1 has been defined as a co-stimulatory receptor that enhances the cytotoxic functions of NK cells. Further investigation showed that it was also found expressed on cytotoxic T CD8^+^ cells, where it maintained its co-stimulatory role [37]. Our results confirmed DNAM-1 expression on both NK and T cells, including CD4^+^ ones, where its function is still unknown. Both baseline and time-dependent models showed an association between an increase in T DNAM-1^+^ cells with a lower probability of eDAEs. Thus, DNAM-1 could be used as a predictive marker for eDAEs emergence.

Other receptors that also showed a significant correlation were PD-1 and CD69 on T cells and PD-1, LAG-3, and CD16 on NK cells. Some of these associations were only found either at baseline or in the time-dependent model. Still, they could be considered as a combined approach to assess the probability of developing eDAEs altogether with DNAM-1 both before and during the treatment.

Whilst DNAM-1 showed the opposite effect on eDAEs depending on its expression on T or NK cells, we analyzed the differences between DNAM-1^+^ cells of each population. On T lymphocytes, DNAM-1^+^ cells had higher expression of PD-1, CD39, CD127, and LAG-3; immune exhaustion markers previously associated with poor HCC outcome [38,39,40]; and higher CD69, a cytotoxicity marker. On the other hand, NK DNAM-1^+^ cells expressed more CD16, an antigen-dependent cytotoxicity receptor with anti-tumor effect in HCC [41], and less LAG-3 and CD39. These results suggest a different role for T and NK DNAM-1^+^ cells: while T DNAM-1^+^ cells have an immune-exhausted phenotype, NK DNAM-1^+^ cells present an activated immune state.

When looking for checkpoint clusters, we found that PD-1 and LAG-3 were associated with CD16 expression on NK-like CD3^+^ cells of patients developing eDAEs, while the correlation was negative in non-eDAEs patients. Moreover, PD-1 correlates with higher eDAEs probability when expressed on NK-like CD3^+^, but it is associated with lower probability when expressed on T cells. These results suggest that specific immune checkpoints such as PD-1 could be involved in opposite cellular processes depending on the cell type where they are expressed. This duality of PD-1 function in NK and T cells has been previously reported in other cancer diseases [27,39,42,43,44].

We then evaluated the expression of the other Ig-like family receptors, CD96 and TIGIT, which are usually co-expressed with DNAM-1 [45]. CD96 was initially defined as an adhesion receptor for T cell immune synapses [46]. However, few studies continued investigating its role until recent years, when it was described as an immune checkpoint inhibitor for NK cells using animal models [23]. At present, we only found one study that has comprehensively investigated the expression of CD96 across human PBMCs, with inconclusive results about its role in T cells [47]. Immunohistochemistry studies have situated CD96 as a marker of poor prognosis and immune exhaustion in HCC [48] as well as in other cancer types like melanoma [49], gastric cancer [28], and pancreatic cancer [50]; some of which also identified TIGIT as a negative prognostic marker.

Our results show that CD96 and TIGIT expression were lower on NK DNAM-1^+^ cells compared to that on DNAM-1^−^ cells, which is consistent with an activated phenotype in NK DNAM-1^+^CD96^−^/TIGIT^−^ cells and an exhausted phenotype in DNAM-1^−^CD96^+^/TIGIT^+^ cells. T cells CD96 expression was significantly higher than that of NK cells, while TIGIT levels were similar. Unlike NK DNAM-1^+^ cells, T DNAM-1^+^ cells had higher CD96 expression than their DNAM-1^−^ counterparts. These results suggest that CD96/DNAM-1 mark a novel phenotype and transcriptional landscape on T cells, with an increase of the immune exhaustion markers PD-1, LAG-3 and CD39 and a decrease of the cytotoxic marker CD69. This is not mirrored by NK DNAM-1/CD96^+^ cells, which have increased expression of cytotoxicity markers (CD16) and less expression of exhaustion markers (LAG-3, CD39). Ex vivo functional assays further support this hypothesis. We show that activated T CD4^+^ DNAM-1^+^ cells release higher amounts of cytokines and suppress the cytotoxic capacity of non-CD4^+^ effector lymphocytes, leading to increased immune suppression.

Lastly, we analyzed Eomes and T-bet expression, transcription factors that play a part in immune cells maturation. We found that T-bet has a significant correlation with DNAM-1 expression across all cell types, suggesting that T-bet is involved in the transcription of DNAM-1, and most likely that of TIGIT and CD96 as well, which is consistent with previous publications [31,32]. However, the amount of T-bet^+^ cells significantly vary amongst the different lymphocyte populations, suggesting that other molecular processes might be involved.

## 4. Materials and Methods

### 4.1. Patients

This prospective study considered 52 HCC patients who initiated sorafenib in our center between June 2016 and March 2019. All the patients provided written informed consent before enrolment. The study was approved by the Institutional Review Board (Ref. HBC/2013/8351 and HBC/2017/0026) and complied with the provisions of the Good Clinical Practice guidelines and the Declaration of Helsinki.

### 4.2. Blood Extraction

Ten mL of blood were collected in BD Vacutainer EDTA K2 tubes (BD Biosciences, 1026367525 Franklin Lakes, NJ, USA). Blood was collected at baseline, 1, 4, and 8 weeks after starting treatment; plus, at all points of treatment modification during the first 8 weeks.

### 4.3. PBMCs Isolation

Peripheral blood mononuclear cells (PBMCs) were isolated from whole blood by density gradient centrifugation using Lymphoprep [Stem Cell Technologies, 07851] as per to manufacturer’s instructions. Briefly, 10 mL of whole blood were diluted with 10 mL of Dulbecco’s phosphate-buffered saline (dPBS) (Sigma-Aldrich, Saint Louis, MI, USA, D8537-500ML) and layered over 10 mL of Lymphoprep; then centrifuged at 800× *g* for 20 min without brake and acceleration. The lymphocyte layer was collected and frozen in 5 million cells aliquots at −80 °C in a solution of 10% dimethyl sulfoxide (DMSO) (Sigma-Aldrich, Saint Louis, Missouri USA, 472301) diluted in fetal calf serum (FCS) (Gibco, Waltham, MA, USA 10082147) until analysis.

### 4.4. Flow Cytometry

Half a million cells corresponding to each patient time-point sample were stained according to previous protocols [51]. For intracellular staining, FOXP3 Transcription Factor Staining Buffer Set (eBioscience, San Diego, California USA 00-5523-00) was used following to manufacturer’s instructions [52]. Blank, single and FMO (fluorescence minus one) controls were included in each assay.

Cell populations labelled with plus or minus signs refer to those cells with (plus) or without (minus) visible expression of the specific markers.

The following lymphocyte cells were identified based on the combined expression of different surface markers (Table S1): T cells, T CD4^+^ cells, cytotoxic T CD8^+^ cells, regulatory T cells (Treg), B cells, Natural Killer (NK) cells, and NK-like CD3^+^ cells. Based on CD56 intensity, NK cells were further grouped into CD56^+bright^ and CD56^+dim^, which have been reported as granulocytic and cytotoxic, respectively. Gating strategy is shown in the Section 2 (Figure 1).

Mean Fluorescence Intensity (MFI) was computed for all parameters in every analysis and normalized over the Sphero™ Rainbow Calibration Particles (8 peaks), 3.0–3.4 µM.

### 4.5. Antibodies and Reagents

All antibodies and reagents used are detailed in Table S2 in the Supplementary Materials.

### 4.6. Cytokine Release Assay

T CD4^+^ cells were isolated from healthy adult buffy coat using the CD4^+^ T Cell Isolation Kit (Miltenyi Biotec, Bergisch Gladbach, Germany, 130-096-533). The non-CD4^+^ fraction was moved into culture for further use in the cytotoxicity assays. The CD4^+^ product was stained using anti-DNAM-1 BV711 for sorting of DNAM-1^+^ and DNAM-1^−^ cells by flow cytometry. A hundred thousand T CD4^+^ cells were then stimulated either with phorbol myristate acetate (PMA) plus ionomycin for 6 h or with plate-bound anti-CD3 and soluble anti-CD28 alone or plus one of the following: IL-15 (PeproTech, Rocky Hill, USA, 200-15, 10 ng/mL), plate-bound anti-CD155 (5 µg/mL), plate-bound anti-CD96 (5 µg/mL), plate-bound anti-DNAM-1 (5 µg/mL), soluble anti-DNAM-1 (20 µg/mL) plus anti-F(ab’)2 (20 µg/mL) or 20.000 inactivated antigen-presenting cells (APCs) for 24 h. IL-2 (PeproTech, Rocky Hill, CT, USA 200-02, 10 ng/mL) was added to all conditions, except to PMA/Ionomycin. For the last 6 h of incubation, Brefeldin-A (500 ng/mL) and anti-CD107a were added. Cells were then fixed and permeabilized and stained for the quantification of IFN-γ, TNF-α, IL-4, IL-10, and Granzyme-B (Grnz-B) by flow cytometry.

### 4.7. Immune-Suppression Assay

T CD4^+^ cells sorted into DNAM-1^+^ and DNAM-1^−^ groups were stimulated for 72 h as described above. PMA/Ionomycin was not used in this assay; instead, sorafenib (10 µM) was added to the cells. The night before the assay 100.000 HuH7 cells were seeded in 24-well plates. After the stimulation T CD4^+^ cells were resuspended in fresh media and 200,000, 100,000, 50,000, or 10,000 cells of each condition were added to the HuH7 cells. Finally, 100,000 autologous non-CD4^+^ cells were added to all wells and incubated for 24 h. After the co-culture, all supernatants were removed, and all wells were washed twice. HuH7 cell viability was assessed using MTS assay according to the manufacturer’s protocol [Abcam, Cambridge, UK ab197010] and cytotoxicity was calculated compared to a HuH7 control culture.

### 4.8. Statistical Analysis

Quantitative variables were expressed as median and interquartile range [IQR 25th–75th percentiles]. Categorical variables were described as absolute frequencies and percentages (%). Comparisons between two groups for quantitative or ordinal variables were assessed by Mann-Whitney U test. Fisher’s exact test was used to compare categorical variables. Paired comparisons were assessed by the signed-rank test for quantitative or ordinal variables, or with McNemar test for categorical variables.

Time to event variables were described using the Kaplan-Meier method, reporting median and confidence intervals (95% CI). The observed survival functions were compared with the log-rank test. Univariate and multivariate Cox regression models were used to estimate Hazard ratios (HR) and their 95% CI.

The following baseline clinically relevant variables were assessed for the multivariate analysis: BCLC status (A–B/C), ECOG PS (0/1), and Child-Pugh Score (A or non-cirrhotic/B). Baseline and time-dependent data were assessed for the lymphocyte populations. The time-dependent data (time-dependent Cox-regression models) consider the evolutionary values of the selected variables at each recorded time. These analyses take into account the changes of specific variables over time to accurately assess their influence on outcome.

All tests were two-sided with *p*-value < 0.05 considered significant. SAS software, version 9.4, was used for all statistical analyses except for the paired differences of DNAM-1^+^/DNAM-1^−^ populations that were calculated and graphically displayed using GraphPad Prism 8.4.1.

## 5. Conclusions

In this work, we explored whether the development of eDAEs was associated with a specific profile in peripheral immune cells. Our study constitutes a proof-of-concept that suggests that this distinct profile may indeed exist. We have reported for the first time the association between baseline differences and evolutionary changes in circulating immune cells in patients with advanced HCC during sorafenib treatment and the probability of developing eDAEs. The development of eDAEs does not depend on a unique immune cell population but on the balance of different immune checkpoints. Since eDAEs are predictors of improved outcome, and our results reinforce the involvement of immune modulation in their development, this strongly suggests that the benefits of sorafenib are, in part, immune-mediated, providing further rationale for its combination with ICI. Additional research on other HCC treatments could show that the correlation between eDAEs, survival, and immunity is not only restricted to sorafenib-treated patients.

## Figures and Tables

**Table 1:** Patient characteristics.

Patients	*N* = 52
Age (Years), median [IQR]	64.34 (56.6–71.9)
Gender (males), *n* (%)	44 (84.6)
Arterial Hypertension (Yes), *n* (%)	28 (53.9)
Diabetes (Yes), *n* (%)	21 (40.4)
Child-Pugh Score (non-Cirrhotic * or A/B)	45 (86.5)/7 (13.5)
Aetiology, *n* (%)	
HCV	13 (25)
Alcohol	14 (26.9)
HBV	3 (5.8)
NASH	4 (7.7)
More than one of previous aetiologies	9 (17.3)
Others	6 (11.5)
Not applicable	3 (5.8)
Previous HCC treatment, *n* (%)	
Liver transplant	3 (5.8)
Surgery	2 (3.9)
Percutaneous	2 (3.9)
Sequential	9 (17.3)
TACE	13 (25)
No previous treatment	23 (44.2)
Ascites (Yes), *n* (%)	7 (13.5)
Encephalopathy (No), *n* (%)	52 (100)
BCLC stage (B/C), *n* (%)	22 (43.3)/30 (57.7)
Vascular Invasion (Yes), *n* (%)	20 (38.5)
ECOG-PS (0/1), *n* (%)	50 (96.1)/2 (3.9)
Alpha-fetoprotein (ng/dL), median [IQR]	17 (8–292)
Extra-hepatic spread (Yes), *n* (%)	22 (42.3)
Haemoglobin (g/L), median [IQR]	13.1 (12.1–14.4)
Prothrombin time (%), median [IQR]	79 (68–90)
Total bilirubin (mg/dL), median [IQR]	0.9 (0.6–1.4)
Conjugated bilirubin (mg/dL), median [IQR]	0.4 (0.3–0.6)
ALT (IU/L), median [IQR]	44 (29–68)
AST (UI/L), median [IQR]	54.5 (38–85)
Alkaline phosphatase (IU/L), median [IQR]	151 (102–193)
GGT (IU/L), median [IQR]	167 (99–265)
Albumin (g/L), median [IQR]	40 (34–43)
Follow-up (months), median [IQR]	9.6 (3.9–19.2)
Treatment time (months), median [IQR]	5.1 (2.5–9.6)
Overall survival (months), median (95% CI)	26.4 (10.7–42.2)
eDAEs (Yes), *n* (%)	16 (30.8)
Overall survival eDAEs (months), median (95% CI)	26.4 (17.4–35.5)
Overall survival non-eDAEs (months), median (95% CI)	16.4 (6.6–NE)
Decompensation (Yes), n (%)	11 (21.2)
Exitus (Yes), *n* (%)	20 (38.5)

NASH: Non-alcoholic steatohepatitis; HBV: Hepatitis B virus; HCV: Hepatitis C virus; TACE: transarterial chemo-embolization; BCLC: Barcelona Clinic Liver Cancer; NE: not estimable; *: 3 non-cirrhotic patients.

**Table 2:** Time-dependent multivariate Cox regression models between B and NK lymphocyte percentages and the probability of developing early dermatologic adverse events (eDAEs) during the first 8 weeks of sorafenib treatment.

Lymphocyte Population	Baseline Adjusting Co-Factors	HR (95% CI)	*p*-Value
B cell	none	1.06 (1–1.11)	0.044
	BCLC stage	1.06 (1–1.12)	0.049
	ECOG-PS	1.06 (1–1.11)	0.051
	Child-Pugh Score	1.05 (1–1.11)	0.06
	BCLC stage|ECOG-PS	1.06 (1–1.12)	0.06
	BCLC stage|Child-Pugh Score	1.05 (1–1.11)	0.06
NK	none	0.91 (0.83–1.01)	0.06
	BCLC stage	0.91 (0.83–1)	0.06
	Child-Pugh Score	0.92 (0.84–1.01)	0.07
	ECOG-PS	0.92 (0.83–1.01)	0.07
	BCLC stage|ECOG-PS	0.92 (0.83–1.01)	0.07
	BCLC stage|Child-Pugh Score	0.91 (0.83–1)	0.055

HR: Hazard Ratio, 95% CI: 95% Confidence Interval.

**Table 3:** Baseline and time-dependent multivariate Cox regression models between immune checkpoints expression in T and NK lymphocytes and the probability of developing eDAEs during the first 8 weeks of sorafenib treatment.

Model	Lymphocyte Population	Baseline Adjusting Co-Factors	HR (95% CI)	*p*-Value
Baseline values	T CD4^+^ DNAM-1^+^	BCLC Stage|Child-Pugh Score|ECOG-PS	0.94 (0.89–0.99)	0.01
	T CD8^+^ DNAM-1^+^	BCLC Stage|Child-Pugh Score|ECOG-PS	0.94 (0.9–0.99)	0.02
	T DNAM-1^+^	BCLC Stage|Child-Pugh Score|ECOG-PS	0.92 (0.87–0.97)	0.003
	NK CD56^+bright^ PD-1^+^	BCLC Stage|Child-Pugh Score|ECOG-PS	0.47 (0.22–0.99)	0.05
	T CD8^+^ CD69^+^	BCLC Stage	1.07 (1–1.14)	0.047
	T CD8^+^ CD69^+^	Child-Pugh Score	1.07 (1–1.14)	0.03
	T CD8^+^ CD69^+^	ECOG-PS	1.07 (1–1.14)	0.04
Time-dependent values	T CD4^+^ DNAM-1^+^	BCLC Stage|Child-Pugh Score|ECOG-PS	0.94 (0.9–0.98)	0.01
	T CD8^+^ DNAM-1^+^	BCLC Stage|Child-Pugh Score|ECOG-PS	0.94 (0.9–0.99)	0.02
	T DNAM-1^+^	BCLC Stage|Child-Pugh Score|ECOG-PS	0.93 (0.89–0.97)	0.002
	NK CD56^+bright^ DNAM-1^+^	BCLC Stage|Child-Pugh Score|ECOG-PS	0.91 (0.85–0.96)	0.002
	NK CD56^+bright^ PD-1^+^	BCLC Stage|Child-Pugh Score|ECOG-PS	0.57 (0.33–0.99)	0.047
	T CD4^+^ PD-1^+^	BCLC Stage|ECOG-PS	0.9 (0.82–0.99)	0.04
	NK-like CD3^+^ CD16^+^	BCLC Stage|Child-Pugh Score|ECOG-PS	1.05 (1–1.08)	0.02
	NK-like CD3^+^ LAG-3 MFI	BCLC Stage|Child-Pugh Score|ECOG-PS	1.06 (1–1.11)	0.05
	NK-like CD3^+^ PD-1 MFI	BCLC Stage|Child-Pugh Score|ECOG-PS	2.11 (1.12–3.95)	0.02

HR: Hazard Ratio, 95% CI: 95% Confidence Interval, MFI: Mean Fluorescence Intensity.

**Table 4:** Correlation models between CD16 positive expression, PD-1 MFI, and LAG-3 MFI quantification in NK-like CD3^+^ cells of eDAEs and non-eDAEs developing patients.

Cell Marker	eDAEs (Yes/No)	LAG-3 MFI	PD-1 MFI
CD16^+^	yes ^$^ no ^&^	r = 0.51 (*p* = 0.1) r = −0.16 (*p* = 0.4)	r = 0.62 (*p* = 0.04) r = −0.15 (*p* = 0.4)
LAG-3 MFI	yes ^$^ no ^&^	- -	r = 0.86 (*p* < 0.001) r = 0.16 (*p* = 0.4)

^$^ n = 11, ^&^ n = 28. MFI: Mean Fluorescence Intensity, eDAEs: early dermatologic adverse events.

## Data Availability

The data presented in this study are available in both article and Supplementary Materials and methods.

## Supplementary Materials

The following are available online at https://www.mdpi.com/2072-6694/13/3/426/s1, Figure S1: Histogram representation of CD69, IL-10 and Grnz-B expression of T CD4^+^ DNAM-1^+^ and DNAM-1^−^ cells after PMA/Ionomycin stimulation, Figure S2: Histogram representation of T cells cytokines expression, Figure S3: Cytotoxic ability of lymphocytes co-cultured with autologous pre-activated T CD4+ cells, Table S1: Definition of the combination of markers used for the identification of every lymphocyte population, Table S2: List of the fluorophores used in the study.

## References

[1] Forner A., Reig M., Bruix J. (2018). Hepatocellular Carcinoma. Lancet.

[2] Mattiuzzi C., Lippi G. (2019). Current Cancer Epidemiology. J. Epidemiol. Glob. Health.

[3] Golfieri R., Bargellini I., Spreafico C., Trevisani F. (2019). Patients with Barcelona Clinic Liver Cancer Stages B and C Hepatocellular Carcinoma: Time for a Subclassification. Liver Cancer.

[4] Finn R.S., Qin S., Ikeda M., Galle P.R., Ducreux M., Kim T.-Y., Kudo M., Breder V., Merle P., Kaseb A.O. (2020). Atezolizumab plus Bevacizumab in Unresectable Hepatocellular Carcinoma. N. Engl. J. Med..

[5] Shrimali R.K., Yu Z., Theoret M.R., Chinnasamy D., Restifo N.P., Rosenberg S.A. (2010). Antiangiogenic Agents Can Increase Lymphocyte Infiltration into Tumor and Enhance the Effectiveness of Adoptive Immunotherapy of Cancer. Cancer Res..

[6] Wang Q., Yu T., Yuan Y., Zhuang H., Wang Z., Liu X., Feng M. (2013). Sorafenib Reduces Hepatic Infiltrated Regulatory T Cells in Hepatocellular Carcinoma Patients by Suppressing TGF-Beta Signal. J. Surg. Oncol..

[7] Zhao W., Gu Y.H., Song R., Qu B.Q., Xu Q. (2008). Sorafenib Inhibits Activation of Human Peripheral Blood T Cells by Targeting LCK Phosphorylation. Leukemia.

[8] Kohga K., Takehara T., Tatsumi T., Ishida H., Miyagi T., Hosui A., Hayashi N. (2010). Sorafenib Inhibits the Shedding of Major Histocompatibility Complex Class I-Related Chain A on Hepatocellular Carcinoma Cells by down-Regulating a Disintegrin and Metalloproteinase 9. Hepatology.

[9] Reig M., Torres F., Rodriguez-Lope C., Forner A., LLarch N., Rimola J., Darnell A., Ríos J., Ayuso C., Bruix J. (2014). Early Dermatologic Adverse Events Predict Better Outcome in HCC Patients Treated with Sorafenib. J. Hepatol..

[10] Díaz-González Á., Sanduzzi-Zamparelli M., Sapena V., Torres F., LLarch N., Iserte G., Forner A., da Fonseca L., Ríos J., Bruix J. (2019). Systematic Review with Meta-Analysis: The Critical Role of Dermatological Events in Patients with Hepatocellular Carcinoma Treated with Sorafenib. Aliment. Pharmacol. Ther..

[11] Rimola J., Díaz-González Á., Darnell A., Varela M., Pons F., Hernandez-Guerra M., Delgado M., Castroagudin J., Matilla A., Sangro B. (2018). Complete Response under Sorafenib in Patients with Hepatocellular Carcinoma: Relationship with Dermatologic Adverse Events. Hepatology.

[12] Bruix J., Merle P., Granito A., Huang Y.-H., Bodoky G., Yokosuka O., Rosmorduc O., Breder V.V., Gerolami R., Masi G. (2018). Hand-Foot Skin Reaction (HFSR) and Overall Survival (OS) in the Phase 3 RESORCE Trial of Regorafenib for Treatment of Hepatocellular Carcinoma (HCC) Progressing on Sorafenib. J. Clin. Oncol..

[13] Zhou X., Yao Z., Yang H., Liang N., Zhang X., Zhang F. (2020). Are Immune-Related Adverse Events Associated with the Efficacy of Immune Checkpoint Inhibitors in Patients with Cancer? A Systematic Review and Meta-Analysis. BMC Med..

[14] Bottlaender L., Amini-Adle M., Maucort-Boulch D., Robinson P., Thomas L., Dalle S. (2020). Cutaneous Adverse Events: A Predictor of Tumour Response under Anti-PD-1 Therapy for Metastatic Melanoma, a Cohort Analysis of 189 Patients. J. Eur. Acad. Dermatol. Venereol..

[15] Rkman D., Likić R., Bebek M., Gnjidić M., Gamulin M. (2019). Skin Autoimmunity Might Be Associated with Increased Efficacy of Atezolizumab in Metastatic Urothelial Carcinoma: A Case Report. Croat. Med. J..

[16] Pardoll D.M. (2012). The Blockade of Immune Checkpoints in Cancer Immunotherapy. Nat. Rev. Cancer.

[17] Ventola C.L. (2017). Cancer Immunotherapy, Part 1: Current Strategies and Agents. Pharm. Ther..

[18] Vaddepally R.K., Kharel P., Pandey R., Garje R., Chandra A.B. (2020). Review of Indications of FDA-Approved Immune Checkpoint Inhibitors per NCCN Guidelines with the Level of Evidence. Cancers.

[19] Anderson A.C., Joller N., Kuchroo V.K. (2016). Lag-3, Tim-3, and TIGIT: Co-Inhibitory Receptors with Specialized Functions in Immune Regulation. Immunity.

[20] Souza-Fonseca-Guimaraes F., Cursons J., Huntington N.D. (2019). The Emergence of Natural Killer Cells as a Major Target in Cancer Immunotherapy. Trends Immunol..

[21] Bryceson Y.T., March M.E., Ljunggren H.-G., Long E.O. (2006). Activation, Coactivation, and Costimulation of Resting Human Natural Killer Cells. Immunol. Rev..

[22] Dougall W.C., Kurtulus S., Smyth M.J., Anderson A.C. (2017). TIGIT and CD96: New Checkpoint Receptor Targets for Cancer Immunotherapy. Immunol. Rev..

[23] Chan C.J., Martinet L., Gilfillan S., Souza-Fonseca-Guimaraes F., Chow M.T., Town L., Ritchie D.S., Colonna M., Andrews D.M., Smyth M.J. (2014). The Receptors CD96 and CD226 Oppose Each Other in the Regulation of Natural Killer Cell Functions. Nat. Immunol..

[24] Zhang H., Vijayan D., Li X.-Y., Robson S.C., Geetha N., Teng M.W.L., Smyth M.J. (2019). The Role of NK Cells and CD39 in the Immunological Control of Tumor Metastases. Oncoimmunology.

[25] Simoni Y., Fehlings M., Kløverpris H.N., McGovern N., Koo S.-L., Loh C.Y., Lim S., Kurioka A., Fergusson J.R., Tang C.-L. (2017). Human Innate Lymphoid Cell Subsets Possess Tissue-Type Based Heterogeneity in Phenotype and Frequency. Immunity.

[26] Park S.L., Gebhardt T., Mackay L.K. (2019). Tissue-Resident Memory T Cells in Cancer Immunosurveillance. Trends Immunol..

[27] Pesce S., Greppi M., Grossi F., Del Zotto G., Moretta L., Sivori S., Genova C., Marcenaro E. (2019). PD/1-PD-Ls Checkpoint: Insight on the Potential Role of NK Cells. Front. Immunol..

[28] He W., Zhang H., Han F., Chen X., Lin R., Wang W., Qiu H., Zhuang Z., Liao Q., Zhang W. (2017). CD155T/TIGIT Signaling Regulates CD8+ T-Cell Metabolism and Promotes Tumor Progression in Human Gastric Cancer. Cancer Res..

[29] Knox J.J., Cosma G.L., Betts M.R., McLane L.M. (2014). Characterization of T-Bet and Eomes in Peripheral Human Immune Cells. Front. Immunol..

[30] Popescu I., Pipeling M.R., Shah P.D., Orens J.B., McDyer J.F. (2014). T-Bet:Eomes Balance, Effector Function, and Proliferation of Cytomegalovirus-Specific CD8+ T Cells during Primary Infection Differentiates the Capacity for Durable Immune Control. J. Immunol..

[31] Li G., Yang Q., Zhu Y., Wang H.-R., Chen X., Zhang X., Lu B. (2013). T-Bet and Eomes Regulate the Balance between the Effector/Central Memory T Cells versus Memory Stem Like T Cells. PLoS ONE.

[32] Gordon S.M., Chaix J., Rupp L.J., Wu J., Madera S., Sun J.C., Lindsten T., Reiner S.L. (2012). The Transcription Factors T-Bet and Eomes Control Key Checkpoints of Natural Killer Cell Maturation. Immunity.

[33] Cibrián D., Sánchez-Madrid F. (2017). CD69: From Activation Marker to Metabolic Gatekeeper. Eur. J. Immunol..

[34] Barata J.T., Durum S.K., Seddon B. (2019). Flip the Coin: IL-7 and IL-7R in Health and Disease. Nat. Immunol..

[35] Xiong P., Sang H.-W., Zhu M. (2015). Critical Roles of Co-Activation Receptor DNAX Accessory Molecule-1 in Natural Killer Cell Immunity. Immunology.

[36] Martinet L., Smyth M.J. (2015). Balancing Natural Killer Cell Activation through Paired Receptors. Nat. Rev. Immunol..

[37] Ramsbottom K.M., Hawkins E.D., Shimoni R., McGrath M., Chan C.J., Russell S.M., Smyth M.J., Oliaro J. (2014). Cutting Edge: DNAX Accessory Molecule 1–Deficient CD8+ T Cells Display Immunological Synapse Defects That Impair Antitumor Immunity. J. Immunol..

[38] Cai X.-Y., Ni X.-C., Yi Y., He H.-W., Wang J.-X., Fu Y.-P., Sun J., Zhou J., Cheng Y.-F., Jin J.-J. (2016). Overexpression of CD39 in Hepatocellular Carcinoma Is an Independent Indicator of Poor Outcome after Radical Resection. Medicine.

[39] Ma J., Zheng B., Goswami S., Meng L., Zhang D., Cao C., Li T., Zhu F., Ma L., Zhang Z. (2019). PD1Hi CD8+ T Cells Correlate with Exhausted Signature and Poor Clinical Outcome in Hepatocellular Carcinoma. J. Immunother. Cancer.

[40] Guo M., Yuan F., Qi F., Sun J., Rao Q., Zhao Z., Huang P., Fang T., Yang B., Xia J. (2020). Expression and Clinical Significance of LAG-3, FGL1, PD-L1 and CD8+T Cells in Hepatocellular Carcinoma Using Multiplex Quantitative Analysis. J. Transl. Med..

[41] Hu J., Wang E., Liu L., Wang Q., Xia D., Bai W., Tie J., Li X., Yuan J., Yang S. (2019). Sorafenib May Enhance Antitumour Efficacy in Hepatocellular Carcinoma Patients by Modulating the Proportions and Functions of Natural Killer Cells. Investig. New Drugs.

[42] Gros A., Robbins P.F., Yao X., Li Y.F., Turcotte S., Tran E., Wunderlich J.R., Mixon A., Farid S., Dudley M.E. (2014). PD-1 Identifies the Patient-Specific CD8^+^ Tumor-Reactive Repertoire Infiltrating Human Tumors. J. Clin. Investig..

[43] Donia M., Kjeldsen J.W., Andersen R., Westergaard M.C.W., Bianchi V., Legut M., Attaf M., Szomolay B., Ott S., Dolton G. (2017). PD-1+ Polyfunctional T Cells Dominate the Periphery after Tumor-Infiltrating Lymphocyte Therapy for Cancer. Clin. Cancer Res..

[44] Hsu J., Hodgins J.J., Marathe M., Nicolai C.J., Bourgeois-Daigneault M.-C., Trevino T.N., Azimi C.S., Scheer A.K., Randolph H.E., Thompson T.W. (2018). Contribution of NK Cells to Immunotherapy Mediated by PD-1/PD-L1 Blockade. J. Clin. Investig..

[45] Kučan Brlić P., Lenac Roviš T., Cinamon G., Tsukerman P., Mandelboim O., Jonjić S. (2019). Targeting PVR (CD155) and Its Receptors in Anti-Tumor Therapy. Cell. Mol. Immunol..

[46] Wang P.L., O’Farrell S., Clayberger C., Krensky A.M. (1992). Identification and Molecular Cloning of Tactile. A Novel Human T Cell Activation Antigen That Is a Member of the Ig Gene Superfamily. J. Immunol..

[47] Lepletier A., Lutzky V.P., Mittal D., Stannard K., Watkins T.S., Ratnatunga C.N., Smith C., McGuire H.M., Kemp R.A., Mukhopadhyay P. (2019). The Immune Checkpoint CD96 Defines a Distinct Lymphocyte Phenotype and Is Highly Expressed on Tumor-Infiltrating T Cells. Immunol. Cell Biol..

[48] Sun H., Huang Q., Huang M., Wen H., Lin R., Zheng M., Qu K., Li K., Wei H., Xiao W. (2019). Human CD96 Correlates to Natural Killer Cell Exhaustion and Predicts the Prognosis of Human Hepatocellular Carcinoma. Hepatology.

[49] Chauvin J.-M., Pagliano O., Fourcade J., Sun Z., Wang H., Sander C., Kirkwood J.M., Chen T.T., Maurer M., Korman A.J. (2015). TIGIT and PD-1 Impair Tumor Antigen-Specific CD8^+^ T Cells in Melanoma Patients. J. Clin. Investig..

[50] Peng Y.-P., Xi C.-H., Zhu Y., Yin L.-D., Wei J.-S., Zhang J.-J., Liu X.-C., Guo S., Fu Y., Miao Y. (2016). Altered Expression of CD226 and CD96 on Natural Killer Cells in Patients with Pancreatic Cancer. Oncotarget.

[51] Support Protocols|BD Biosciences-US. https://www.bdbiosciences.com/us/resources/s/cellsurface.

[52] BestProtocols: Staining Intracellular Antigens for Flow Cytometry-ES. //www.thermofisher.com/es/es/home/references/protocols/cell-and-tissue-analysis/protocols/staining-intracellular-antigens-flow-cytometry.html.

