# Supplementary material for: Activated Lymphocytes and Increased Risk of Dermatologic Adverse Events during Sorafenib Therapy for Hepatocellular Carcinoma
Source: Cancers (Basel). 2021 Jan 23;13(3):426. doi: 10.3390/cancers13030426 (PMC7865624; doi:10.3390/cancers13030426)
Supplement: Supplementary file 1 [file cancers-13-00426-s001.pdf]

cancers

MDPI

Supplementary Materials

Activated Lymphocytes and Increased Risk of Dermatologic Adverse Events during Sorafenib Therapy for Hepatocellular Carcinoma

Josep Corominas, Victor Sapena, Marco Sanduzzi-Zamparelli, Cristina Millán, Esther Samper, Neus Llarch, Gemma Iserte, Ferràn Torres, Leonardo G. Da Fonseca, Sergio Muñoz-Martinez, Alejandro Forner, Jordi Bruix, Loreto Boix and María Reig

Table S1. Definition of the combination of markers used for the identification of every lymphocyte population.

Population	Subpopulation	Combination
T		$CD3^{+}CD56^{-}$
	T $CD4^{+}$	$CD3^{+}CD56^{-}CD4^{+}CD8^{-}$
	T $CD8^{+}$	$CD3^{+}CD56^{-}CD8^{+}CD4^{-}$
	Treg	$CD3^{+}CD56^{-}CD4^{+}CD8^{-}CD25^{+}CD127^{-}$
B		$CD3^{-}CD56^{-}CD19^{+}$
NK		$CD56^{+}CD3^{-}$
	$CD56^{+bright}$	$CD56^{+bright}$ $CD3^{-}$
	$CD56^{+dim}$	$CD56^{+dim}$ $CD3^{-}$
NK-like $CD3^{+}$		$CD56^{+}CD3^{+}$
	$CD56^{+bright}$	$CD56^{+bright}$ $CD3^{+}$
	$CD56^{+dim}$	$CD56^{+dim}$ $CD3^{+}$

T: T cells, T CD4+: T helper cells, T CD8+: cytotoxic T cells, Treg: Regulatory T cells, NK: Natural Killer cells.

Table S2. List of the fluorophores used in the study.

Marker	Fluorophore	Reference
CD3	Pacific Blue	BioLegend, 300431
CD4	BUV395	BD Biosciences, 563550
	AlexaFluor700	BD Biosciences, 557922
CD8	Brilliant Violet 605	BioLegend, 344742
CD19	Brilliant Violet 711	BioLegend, 302246
	PE	eBioscience, 12-0199-42
CD56	APC	eBioscience, 17-0566-42
	BUV395	BD Biosciences, 563554
CD25	PE	BioLegend, 356134
	Brilliant Violet 785	BioLegend, 302638
CD16	Brilliant Violet 785	BioLegend, 302046
PD-1	FITC	BioLegend, 369310
CD69	PerCP	BioLegend, 310928
CXCR6	Brilliant Violet 510	BD Biosciences, 743598
LAG-3	PE-Cy7	BioLegend, 369310
CD127	APC-eFluor780	eBioscience, 47-1278-42
CD39	Brilliant Violet 510	BioLegend, 328219

NKG2D	Alexa Fluor 700	R&D Systems, FAB139N-100
DNAM-1	Brilliant Violet 605	BioLegend, 338323
	Brilliant Violet 711	BioLegend, 338334
	Purified	BD Biosciences, 559786
TIGIT	Brilliant Violet 605	BioLegend, 372172
CD96	BB515	BD Bioscience, 564774
	Purified	HyCult Biotech, HM2210-100UG
Eomes	PE-eFluor 710	eBioscience, 61-4877-42
T-bet	eFluor660	eBioscience, 50-5825-80
Viability	VivaFix 353/442	BioRad, 1351111
IL-10	eFluor 660	eBioscience, 50-7108-42
IL-4	PE	eBioscience, 12-0086-41
IFN-γ	Alexa Fluor 488	eBioscience, 53-7319-41
TNF-α	Brilliant Violet 785	BioLegend, 502947
Granzyme B	PE/Cyanine7	BioLegend, 372213
CD107a	Brilliant Violet 605	BioLegend, 328633
CD155	Purified	ThermoFisher, MA5-13493
IgG F(ab')2	Purified	ThermoFisher, 31192

In addition, FcR blocking reagent [Milteny Biotech, 130-059-901] was added to all stainings. Sphero™ Rainbow Calibration Particles (8 peaks), 3.0 - 3.4 μm [BD Biosciences, 559123] were acquired after each assay for control calibration.

Staining buffer was composed of dPBS supplemented with 2% FBS and 1mM EDTA.

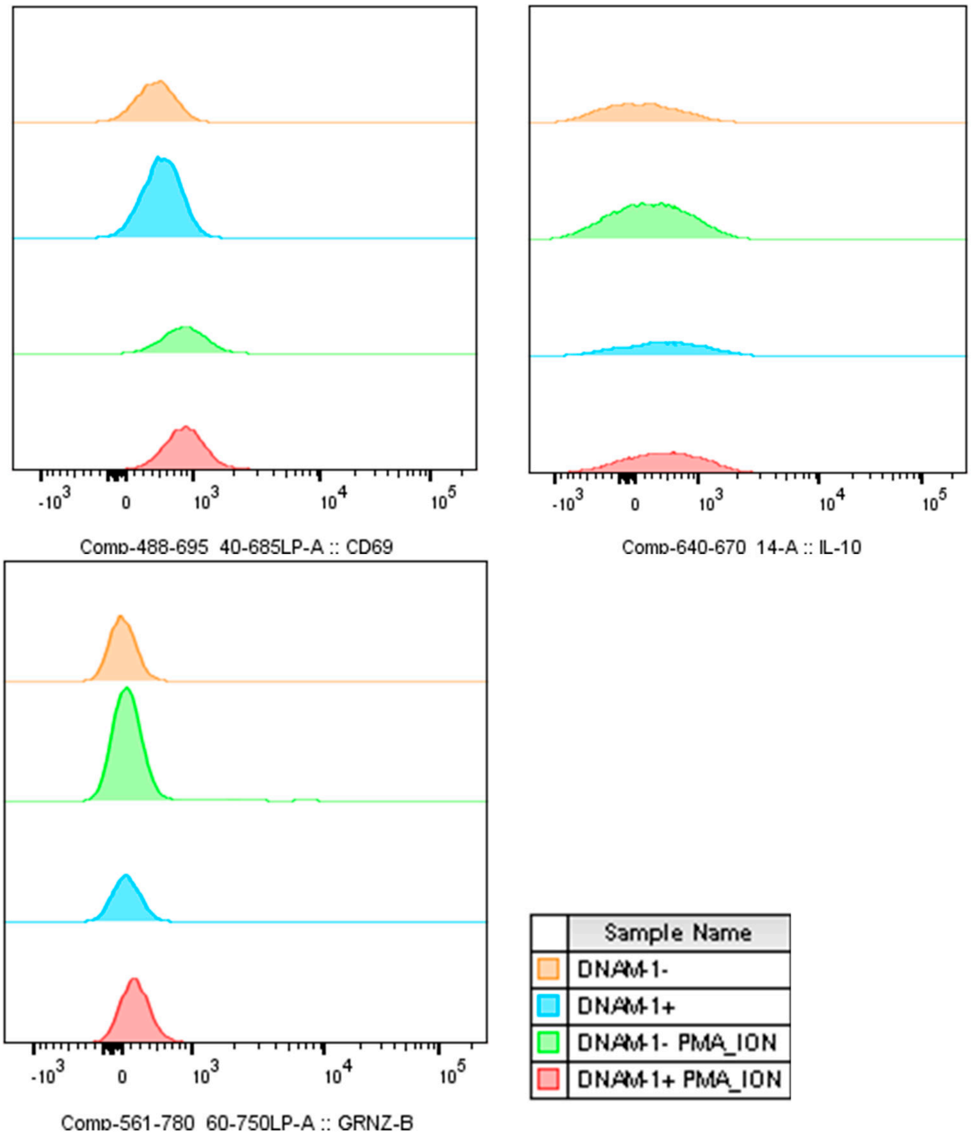

Figure S1. Histogram representation of CD69, IL-10 and Grnz-B expression of T CD4+ DNAM-1+ and DNAM-1− cells after PMA/Ionomycin stimulation. Histogram representation of CD69, IL-10 and Grnz-B expression of T CD4+ DNAM-1+ and DNAM-1- cells with or without PMA/Ionomycin stimulation. Grnz-B: Granzyme B, PMA: phorbol myristate acetate, ION: Ionomycin.

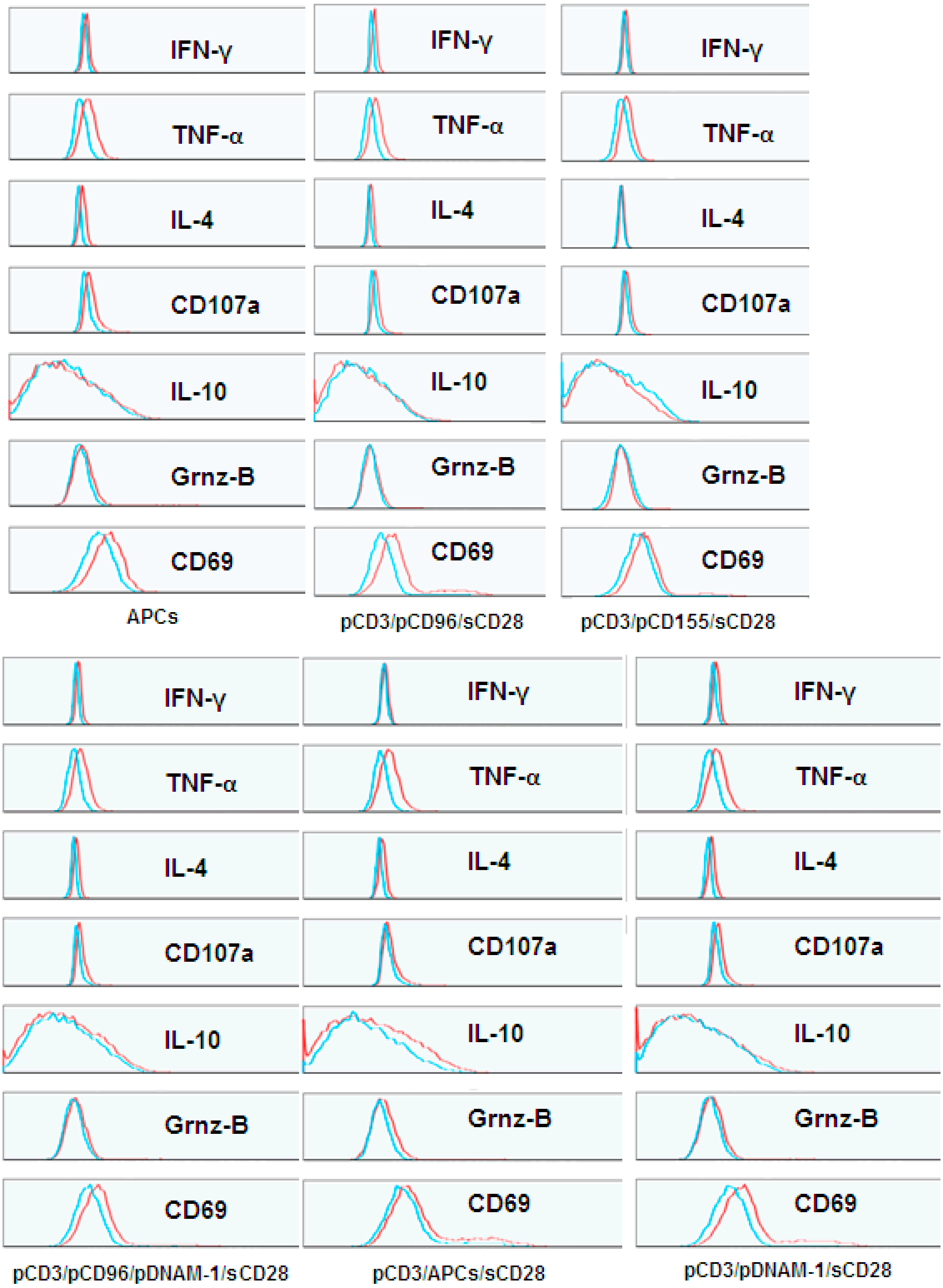
IFN-γ
TNF-α
IL-4
CD107a
IL-10
Grnz-B
CD69
APCs
pCD3/pCD96/sCD28
pCD3/pCD155/sCD28
pCD3/pCD96/pDNAM-1/sCD28
pCD3/APCs/sCD28
pCD3/pDNAM-1/sCD28

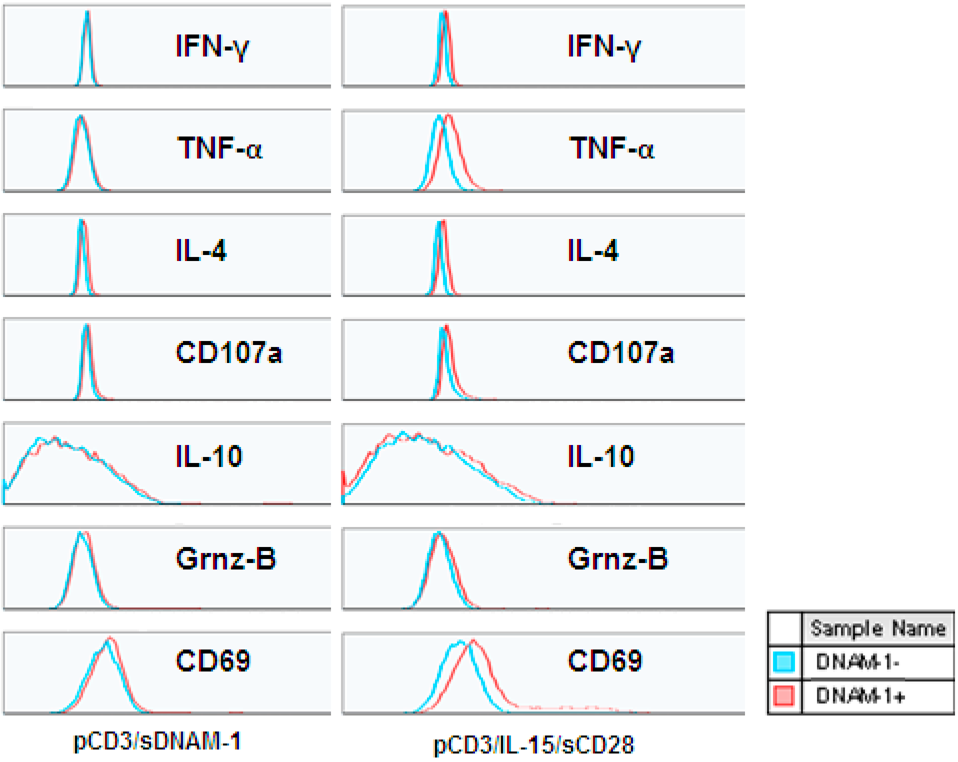

Figure S2. Histogram representation of T cells cytokines expression. T CD4+ DNAM-1+ and DNAM-1- cells were activated for 72h using different combinations of the following: plate-bound CD3, CD155, CD96, DNAM-1 and soluble CD28, DNAM-1 and IL-15. Then, we evaluated the expression of IFN-γ, TNF-α, IL-4, CD107a, IL-10 and Grnz-B. pCD3: plate-bound anti-CD3, sCD28: soluble anti-CD28, pDNAM-1: plate-bound anti-DNAM-1, pCD155: plate-bound anti-CD155, pCD96: plate-bound anti-CD96.

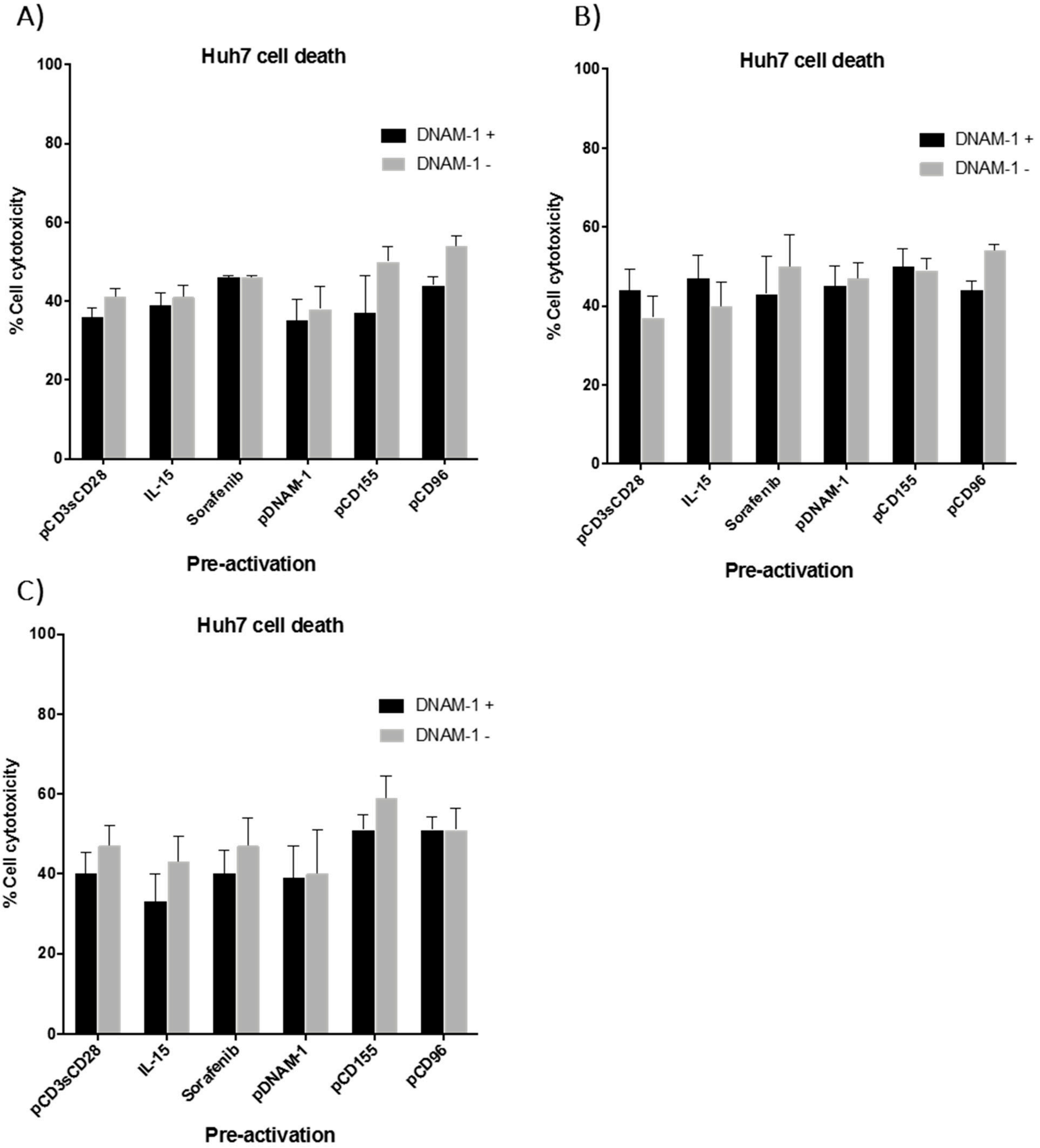

Figure S3. Cytotoxic ability of lymphocytes co-cultured with autologous pre-activated T CD4+ cells. Cytotoxic capacity of CD4- effector lymphocytes co-cultured with autologous pre-activated T CD4+ cells, either DNAM-1+ or DNAM-1-, at ratios 1:1 (A), 1:2 (B) and 1:10 (C). pCD3: plate-bound anti-CD3, sCD28: soluble anti-CD28, Sora: Sorafenib, pDNAM-1: plate-bound anti-DNAM-1, pCD155: plate-bound anti-CD155, pCD96: plate-bound anti-CD96.
